# Critical Hazards Identification and Prevention of Cascading Escalator Accidents at Metro Rail Transit Stations

**DOI:** 10.3390/ijerph17103400

**Published:** 2020-05-13

**Authors:** Zhiru Wang, Ran S. Bhamra, Min Wang, Han Xie, Lili Yang

**Affiliations:** 1 School of Economics and Management, University of Science and Technology Beijing, Beijing 100083, China; xiehan@xs.ustb.edu.cn; 2 School of Business and Economics, Loughborough University, Loughborough LE11 3TU, UK; 3 School of Mechanical, Electrical and Manufacturing Engineering, Loughborough University, Loughborough LE11 3TU, UK; r.s.bhamra@lboro.ac.uk; 4 Department of Safety Supervision, Beijing Subway Limited, Beijing 100044, China; wangmin5895@bjsubway.com

**Keywords:** metro rail transit station, cascading escalator accident, task-driven method, complex network theory

## Abstract

Escalator accidents not only happen frequently but also have cascading effects. The purpose of this study is to block the formation of cascading accident networks by identifying and preventing critical hazards. A modified five-step task-driven method (FTDM) is proposed to break down passenger-related cascading escalator accidents. Three complex network parameters in complex network theory are utilized to identify critical and non-critical Risk Passenger Behavior (RPB) hazards and Other Hazards related with Risk Passenger Behavior (OH-RPB) in accident chains. A total of 327 accidents that occurred in the Beijing metro rail transit (MRT) stations were used for case studies. The results are consistent in critical and non-critical RPB and OH-RPB and prove that through combination of FTDM accident investigation model and complex network analysis method, critical and non-critical RPB and OH-RPB in a complicated cascading hazards network can be identified. Prevention of critical RPB can block the formation of cascading accident networks. The method not only can be used by safety manager to make the corresponding preventive measures according to the results in daily management but also the findings can guide the allocation of limited preventive resources to critical hazards rather than non-critical hazards. Moreover, the defects of management plan and product design can be re-examined according to the research results.

## 1. Introduction

Stairs, escalators, and elevators are the three ways by which passengers access subways globally. The most popular choice of these is riding escalators [1]. However, escalator accidents comprise the largest percentage of all injuries and deaths happening on special equipment in China [2] and the Hong Kong metro rail transit (MRT) [3]. Between 2013 and 2015, there were a total of 950 escalator accidents reported by the Guangzhou MRT [4]. Escalator-related accidents frequently account for more than 50% of all station accidents in the Hong Kong MRT [3].

Due to the multi-disciplinary operational characteristics of subway systems, as well as exogenous and endogenous functional dependencies and interdependencies, accidents happening on escalators are not only frequent but can also trigger cascading disruptions [5]. Once a passenger falls, a cascading crush may be caused, such as that which occurred at the Beijing Zoo station of the Beijing MRT in 2014 (one death, more than 28 injuries). Hazards can mutually interact, due to the spatial limitations and operational characteristics of MRT systems [6]. The term hazard means behaviors, phenomena, or processes that can induce negative impacts, as defined in [7]. Correspondingly, the term hazard interaction is defined as a direct causative relationship between any two hazards [7]. A hazard could act as the trigger for another hazard [5,8] but may not be the only cause. These hazard interactions and hazards can be said to form a Cascading Escalator Hazards Network (CEHN) [7]. In complex cascading accident networks, it makes sense to identify critical and non-critical hazards. As how to allocate the limited resources in these critical rather than non-critical hazards is an important work of safety management.

Traditional methods of accident investigation lack of detailed data on the incidents makes it impossible to draw sensible conclusions and implement sensible countermeasures [1,4]. An efficient investigation model should be proposed for breaking down these passenger-related cascading escalator accidents. Moreover, the literature on escalator accidents typically uses “single hazard” methods, in which a hazard is generally treated as one particular phenomenon [7]. Such approaches are based on the hypothesis that hazards are independent or isolated [7]. Other related hazards or risks may be increased or underestimated by this lack of a holistic approach. Hence, capturing the holistic characteristics of CEHNs is essential for disaster management in MRT escalator operations. In this context, this paper aims to investigate a comprehensive set of interactions among hazards in MRT systems. We investigated 799 MRT escalator accidents, of which 327 were valid and complete. After extracting the relationships among hazards, a CEHN was formed and the topological characteristics of the CEHN analyzed by utilizing complex network theory. The remainder of this paper is organized as follows: In Section 2, a literature review about the safety analysis of escalators in general is presented, and the drawbacks of the current research are discussed. The process of forming our CEHN is presented in Section 3. Section 4 utilizes complex network theory to explore the statistical properties of the proposed CEHN. Finally, based on the results of Section 4, system improvement strategies are discussed in Section 5.

## 2. Literature Review

Despite the frequency of escalator accidents at subway stations, there have been few studies on escalator-related accidents. The literature on escalator accidents can be categorized into three groups, according to the aim of the research. The first group focuses on exploring the kinds of behavior that passengers have that are significantly correlated with specific escalator accidents and the contribution of operation factors to accidents [1,4]. Such studies are often based on MRT escalator accidents. The second group focuses on describing the epidemiology of escalator-related injuries [9,10,11,12,13,14] by using data from hospitals. The characteristics of passengers, such as gender, age, and health status, are of interest to these researchers. These two groups both approach the topic from a statistical perspective. The last group focuses on the process of the evolution of escalator accidents in a virtual world by utilizing simulation methods.

From the perspective of epidemiology of escalator-related injuries, most studies have focused on children and old adults. The trends of gender, age, hazard type, injured body part(s), and type of injury in adults aged 65 and older were studied [13]. They found that the rate of head injuries and the rate of hospitalization increased with age. Escalator accidents can result in severe trauma in patients aged 16 and older [15]. Significant gender differences in escalator accidents have been observed. Alcohol intoxication and age are significant risk factors in escalator accidents. Other studies have focused on children [9,11]. However, an epidemiological database alone cannot guide the safety manager in solving the safety problems it uncovers. For solutions, analysts must examine the etiology of accidents and must understand the detailed sequence of hazards that culminate in accidental injury [16].

Therefore, statistical and simulation methods have been used to explore the extended contribution factors for escalator accidents. From a passenger behavior hazard perspective, 950 accidents were analyzed to identify the characteristics and risk factors associated with escalator-related injuries in China [4]. The authors concluded that failing to stand firmly was the main cause of escalator-related injuries in elderly passengers, with other major causes including passengers doing other things, not holding handrails, and unwell passengers. Factors such as casualties, tasks, products, and environmental and relationships between major cause and casualty characteristics were analyzed in the Taipei MRT system [1]. It was found that the major accident-causing behaviors of passengers were not holding the handrail and riding improperly (e.g., playing, walking, running, or sitting on the escalator) [9]. It was found that walking on a moving escalator was the main cause of injury in people under age 65 and suggested that young passengers should be prevented from walking on escalators [14].

Additionally, simulation methods have been utilized to study the evolution process of escalator accidents in a virtual environment. Li et al. used simulation tools to study the impact of key factors on group stampede risk during escalator transfer [17]. They concluded that the propagation speed of the accident was always faster than the recovery rate and that taking emergency measures in a timely manner can reduce the severity of the accident. The impact of contributing factors on individual stampede probability was analyzed, and four factors with the highest impact were determined: pedestrian traffic, picking-up duration, pedestrian velocity, and escalator velocity (in descending order) [18]. The practical capacity of escalators has been calculated using a simulation based on passenger behavioral rules [19]. The authors found that prohibiting walking on escalator can improve the capacity (especially in emergency scenarios) by reducing variability in the escalator system.

These studies mentioned above all attempted to study the contribution of hazards to escalator accidents through linear or simulation methods, in order to formulate accident management plans. These techniques have traditionally focused more on single hazards while ignoring the dynamic interactions between hazards originating from escalator equipment, passengers, the surrounding environment, and maintenance in the escalator accidents. However, according to Heinrich’s Law [20], casualty accidents are not the result of an isolated hazard, but the result of a series of hazards [21,22]. In MRT escalator system, accidents are much more likely to be caused by multiple hazards, rather than a single hazard [2]. There may be a causal relationship between these hazards; that is, consequent hazards are triggered by causality hazards. The dynamic interactions between hazards usually constitute multiple interaction loops to form a complex network structure. As such trigger networks exist between accidents, we may be able to defeat the whole accident network by controlling some key hazards, which may be thought of as the Achille’s Heel of the network. On the contrary, due to the accessibility characteristics of the network, even eliminating some hazards may not play a positive role in controlling the whole accident network. Therefore, there is a need to rethink the methodology by exploring the actual dynamic interactions of hazards in escalator accidents from a holistic view.

Accordingly, a combination of the Interpretive Structural Modeling (ISM) and Decision Making Trail and Evaluation Laboratory (DEMATEL) methods was utilized in [2] to establish a hierarchical structure of the hazards, considering the four aspects proposed by [23]. Although this was a big step forward in transferring the research perspective from single hazard analysis to holistic analysis, the interactions between the hazards were determined by experienced experts who understand the inter-relationships and degrees of importance of all hazards. Thus, it is a subjective method which is more suitable in case of lack of data. In the face of a large range of systems, the method is difficult to use, as the complicated matrices required make it difficult for experts to provide reasonable judgment. Additionally, the network established by ISM and DEMATEL is not the actual interaction network of the hazards.

Due to the aforementioned reasons, the aim of this research is to provide an objective method by using large amount of accident data to explore a holistic picture of the actual dynamic interactions between hazards from escalators, passengers, the environment, and maintenance in escalator accidents. Based on dynamic characteristic analysis, we determine the key hazards and hazard interactions which the network is vulnerable or robust to. Complex network theory, which has emerged in recent years, offers new insights in revealing the topological characteristics of complex interactions. By abstracting highly interconnected units into graphs, whose nodes represent the units and whose edges stand for the interactions between them, complex network theory can capture the global properties of the system in a more quantitative and physical way. A subway operation hazard network (MOHN) was established to explore the scale-free and small-world characteristics among hazards [24]. The topological characteristics of a subway construction accident network (SCAN) were analyzed using complex network theory, in order to understand the complexity of subway accidents. Network modeling approach to identify the critical and vulnerable modules in subway systems was proposed [25]. With the assistance of network theory, the inherent features of the complex system, such as degree, node cluster coefficiency (CC), and betweenness of nodes, are made easily available for analysis [26]. Applying complex network theory to explore the topological features of CEHN, it is far easier to recognize the critical hazards and, therefore, accurate and efficient countermeasures can be accordingly derived.

It can be observed, that most existing studies treated cascading escalator accidents generally as one particular phenomenon. They are far from sufficient, in terms of considering hazards from escalators, passengers, the environment, and maintenance in escalator accidents as a dynamic interactions network. The dynamic interactions between hazards usually constitute multiple interaction loops to form a complex network structure. Other related hazards or risks may be increased or underestimated by this lack of a holistic approach. Hence, capturing the holistic characteristics of CEHNs is essential for disaster management in MRT escalator operations. Therefore, the aim of this study is to explore the key hazards and hazard interactions, to which the network is vulnerable or robust, by utilizing complex network theory.

## 3. Methods

### 3.1. Cascading Escalator Accidents Investigation Methods

It was found that the present accident record format is insufficient in analyzing CEHN. The detailed sequences of hazards that culminated in the accidents were not clear. The Drury and Brill 4-step investigation models had the most useful procedures for determining the appropriate level of breakdown of the sequences of hazards. At the start of the investigation (or the sequence breakdown), it should be known what action was intended by the casualties before the accident, then what went wrong with these intentions, why recovery or avoiding action by the casualties was not taken, and finally what pattern of interaction formed between the casualties and the escalator. Based on this, the Drury and Brill 4-step investigation model was modified into a modified five-step task-driven method (FTDM). The difference between Drury and Brill 4-step investigation models is that Step 4 was added into FTDM. The reason for this is that no matter what kind of (i.e., whether or not) recovery or avoiding action the casualties had taken in step 3, the last action (passive behavior) acted by the casualties exists in every accident before the interaction. For example, in one case, the casualties finally fell on the escalator, which was the pattern of interaction between the casualties and the escalator. The falling could have been the result of loss of balance or being knocked over by objects or other passengers. A description of the last passive behavior acted by the casualties can clarify the sequence of hazards. Accordingly, the FTDM asks for reconstruction of the sequence of hazards, as represented by the following steps:

Step 1. Action intended by casualties immediately before the accident: What were they trying to do before anything went wrong? For example, in a fall accident on the escalator, we want to know whether the casualties were standing on an escalator and reading the poster warning signs about facilities in the station before falling or carrying a suitcase.

Step 2. At the moment the goal could not be completed as intended: What went wrong with these intentions? For example, we need to know the fall occurred due to the speed changes, such that they fell backward down the stairs due to inertia.

Step 3. At the moment they took a new, perhaps corrective action but before the accident: Why were not recovery or avoidance actions taken by the casualties, or why did they not work? We need to know whether casualties reached for the handrail, and why it did not work if they did; for example, they reached, but could not really hold it because it was too crowded.

Step 4. The last action the casualties were doing before the accident happened. No matter what kind of recovery or avoidance action the casualties had taken, a last action—the passive behavior acted by the casualties—exists before every accident. As mentioned above, the passive behavior is acted out by the casualties; for a fall, it can be loss of balance, or being knocked over by objects or by passengers.

Step 5. At the moment of the accident: How was energy transferred to cause the injury? What was the pattern of interaction between the casualties and the escalator? We want to know which body part contacted which part of the escalator. Continuing with our example, the passenger fell, and the back of their head made contact with the sharp metal edge of the escalator stair.

### 3.2. Network Modelling Methods

**Figure 1:**
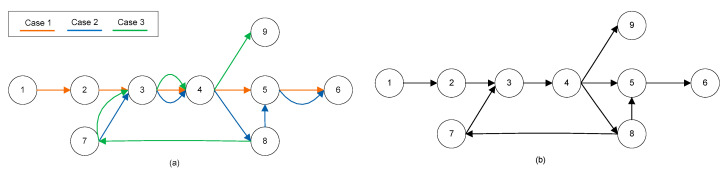
(**a**) A simple escalator accident map. Hazards connected in the map happened in three sample cases; (**b**) L-space graph. The hazard chain of the Case 1 is composed by hazards (nodes) 1,2, 3,4,5, and 6; the hazard chain of the Case 2 is composed by hazards (nodes) 7,3,4, 8,5, and 6; the hazard chain of the Case 3 is composed by hazards (nodes) 8,7,3, 4, and 9.

A straightforward representation of an escalator accident map in the form of a graph represents every hazard by a node, while the edges correspond to the relationships that exist between hazards in each accident. The accident network composed by three sample cases is represented in Figure 1a, as an example. A simple graph to represent this situation is firstly introduced; see Figure 1b. The form of this representation was defined as an L-space in [27]. This graph represents each hazard by a node, and an edge between two nodes indicates that there was at least one accident chain that had the two hazards consecutively (with direction). No multiple edges are allowed. Repeated connections are represented by weights between two hazards.

There are several visualization tools to manifest the structure of a complex network, such as Pajek, Ucinet, and Graphi [28]. The VOS Viewer software [29] is among the most popular computer software including various visualizing techniques. In this paper, the VOS Viewer software was employed to visualize Beijing CEHN. Exploring the network structure by calculation is much more concise and precise than by visual inspection. Therefore, Pajek was employed to assist in analyzing the corresponding topological characteristics of degree and strength, un-weighted clustering coefficient (CC), and betweenness of nodes in this study. Weighted CC of hazards and betweenness of events were calculated using the simulation system called Urban-metro-cas [30], which was developed by the author of this research. The definition and mathematical description of degree, clustering coefficient, and betweenness are described in Section 3.3 (Network analysis methods part). There are eight types of formats for representing a network data file, such as Pajek networks, Pajek matrices, Vega graphs, GEDCOM, UCINET DL, Ball and Stick, Mac Molecule, and MDL MOL [25]. The Pajek matrix format was applied to the network input file in this study. The output network file of Pajek could be read by VOS Viewer for visualization.

### 3.3. Network Analysis Methods

Many studies have shown that the topology of a network plays a crucial role in determining the network’s dynamic features [31]. Exploring the network structure by calculation is much more concise and precise than visual inspection. Calculating the topological parameters can be of benefit in analyzing accidents spreading in a bound sense and capturing the full complexity of a CEHN. Three parameters—degree distribution, clustering coefficient, and betweenness centrality—are explored in this section. Network analysis was carried out from weighted and un-weighted perspective. Un-weighted network is used to describe the topological relationship of hazards in the network. While weighted network is used to describe the impact of events frequency. The frequency of specific relationships with hazards (describing the edges of the graph) is considered as weight on edge. Degree and strength of a node are both indicators used to describe the adjacencies of a hazard. The CC in un-weighted and weighed networks is a measure of the degree to which hazard in a CEHN tend to cluster together. Betweenness centrality is used to find hazards or events that serve as bridges from one part of a graph to another in the topological structure. The definitions and mathematical descriptions of the above indicators are introduced in the following.

#### 3.3.1. Degree and Strength of Hazards

• The Degree of a Hazard

The degree of a hazard is an indicator that describes the adjacencies of a hazard within an un-weighted CEHN in L-space. In the directed CEHN, the degree of a hazard has two components: in-degree and out-degree. The hazard degree is defined according to Formula (1), which represents the number of neighbors that a hazard has; that is, the higher the degree of the hazard, the more connected it is and the more importance it has in the network from the topological structure perspective [32].

(1) $$k_{i}=\sum_{j\in N}a_{ij}$$

where $k_{i}$ is the degree of hazard i, and $a_{ij}$ corresponds to the edges connected to the hazard. These edges take a value of one if hazard i has a track leading in or out.

• The Strength of a Hazard

Hazards degree has generally been extended to the sum of weights when analysis weighted network and labelled hazard strength. In the directed CEHN, the strength of a hazard has two components as well: in-strength and out-strength. The measure has been formalized as follows:

(2) $$s_{i}=\underset{j\in N}{\sum }w_{ij}$$

where w is the weighted adjacency matrix, in which $w_{ij}$ is greater than 0 if the hazard i is connected to hazard j, and the value represents the frequency of event, which is hazard i to hazard j. The degree of hazards is a special case when the hazard strength is equal to 1.

#### 3.3.2. The CC of Hazards

• The Un-Weighted CC

The un-weighted CC is a measure of the degree to which nodes in a graph tend to cluster together. In other words, the hazard that has higher clustering means that its neighbor hazards can easily arrive to each other because of redundant edges among neighborhoods from a topological perspective. The directions of the arcs in the CEHN are not considered when calculating the CC. The clustering degree is based on triplets of hazards. A triplet is three nodes that are connected by either two (open triplet) or three (closed triplet) edges [33]. A triangle graph, therefore, includes three closed triplets, one centered on each of the nodes [34]. In other words, the clustering of node i calculated the total number of existing edges within the neighborhood k(i), say e(i), to the maximum number of edges that could exist, $e{\left(i\right)}^{max}=k\left(i\right)\left(k\left(i\right)-1\right)/2$ (in the case of all the vertices of the neighborhood were connected with one another) [35].

(3) $$c{\left(i\right)}^{un-w}=\frac{e\left(i\right)}{e{\left(i\right)}^{max}}=\frac{2\cdot e\left(i\right)}{k\left(i\right)\left(k\left(i\right)-1\right)}$$

The global un-weighted CC of a network is calculated as the average of the clustering of n nodes:

(4) $$C^{un-w}=\frac{1}{n}\underset{i}{\sum }c{\left(i\right)}^{un-w}$$

• The Weighted CC

The weighted CC is the expansion of the un-weighted CC from the frequency of events. It is used to describe cluster ability of hazards from actual perspective but not only from the topological structure perspective. The frequency of events (edges from hazard to hazard) is the actual attribute of CEHN. Therefore, contributions of three events (edges) to the weighted CC of the hazard in a triangle graph is proportional to the weight of the edges. In order to control the weighted CC of hazards into [0,1], normalized weight of events $\tilde{w_{ij}}$ is used which refers to weight of events $w_{ij}$ dividing by the maximum weight max(w) in the network.

(5) $$c{\left(i\right)}^{w}=\frac{2}{k\left(i\right)\left(k\left(i\right)-1\right)}\underset{j,k}{\sum }\tilde{w_{ij}}\cdot \tilde{w_{jk}}\cdot \tilde{w_{ik}}$$

The global weighted CC of a network is calculated as the average of the clustering of n nodes:

(6) $$C^{w}=\frac{1}{n}\underset{i}{\sum }c{\left(i\right)}^{w}$$

#### 3.3.3. The Betweenness of Hazards

Betweenness centrality is often used to find nodes or edges that serve as bridges from one part of a graph to another [32]. Therefore, frequency of events is not taken into account when calculating betweenness. Betweenness is considered as an important tool in safety management. In an accident network, if a bridge is broken, all OD pairs [33] of hazards cannot be accessed. The betweenness centrality of node or edge is calculated by the ratio of the number of shortest paths passing through this node or edge to all shortest paths between all OD pairs in the network:

(7) $$B(i)=\sum_{j.k\neq i}\frac{p_{jk}(i)}{p_{jk}}$$

where B(i) is the betweenness of node i, $p_{\mathrm{jk}}$ is the total number of shortest paths between node j and k in the network, and $p_{jk}\left(i\right)$ is used to describe the number of shortest paths between node j and k that pass through node i. A path length means how many nodes an accident goes through, and the shortest path refers to the path that passes through the least number of nodes. The value of the betweenness is between 0 and 1. The higher the betweenness, the more important the hazards or hazard interactions between hazards are in the CEHN.

## 4. Case Study

### 4.1. Beijing Cascading Escalator Accidents Data Gathering

**Figure 2:**
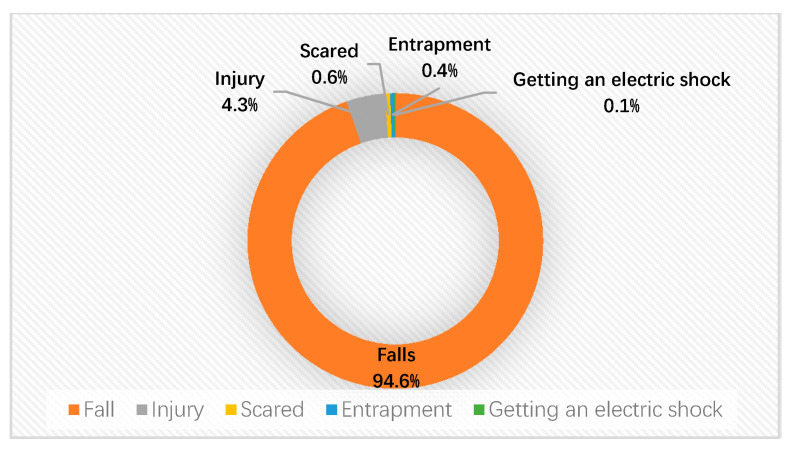
Statistics of accident types happened in Beijing metro rail transit (MRT) escalator.

The accident data was provided by Beijing Subway. There were an estimated 895 casualties involved in 799 escalator accidents in the Beijing MRT stations reported during the period between 2016 and 2018. Of all accidents, 58.3% of the casualties were female, and 35.35% of them were male. Accident types included entrapment, falling, electric shock, injury, and scared (see Figure 2). Falls accounted for the largest proportion of the five types, with a value of 94.6%. Injury followed, accounted for 4.3%. The proportions of the other hazards were less than 5%.

### 4.2. Beijing CEHN Modelling and Visualization

Based on FTDM, 472 accidents that did not have a process description or only had one hazard were excluded. Thus, there were 327 accidents that were valid and complete in the final sample. Of the 327 accidents, 61 hazards were identified from escalator, maintenance, environment, and passenger perspectives (see Table 1). As a result, a total of 327 accident were obtained, as presented in Table 2.

**Figure 3:**
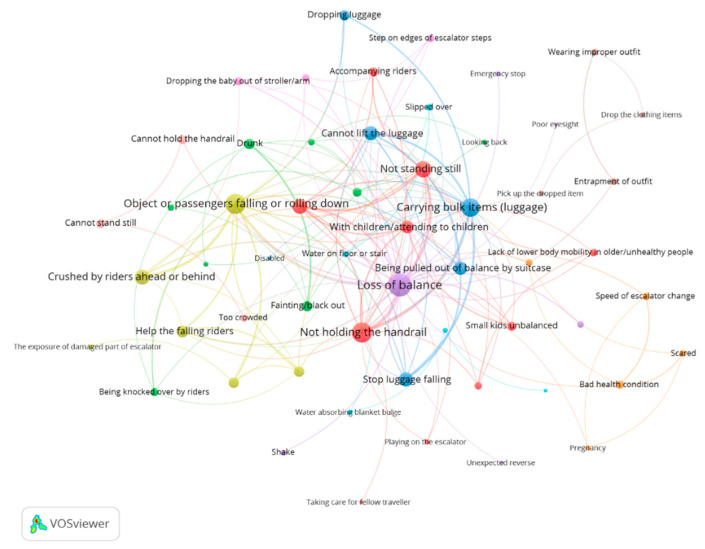
Map (Label view) of a partial collection of hazards in the Cascading Escalator Hazards Network (CEHN).

**Figure A1:**
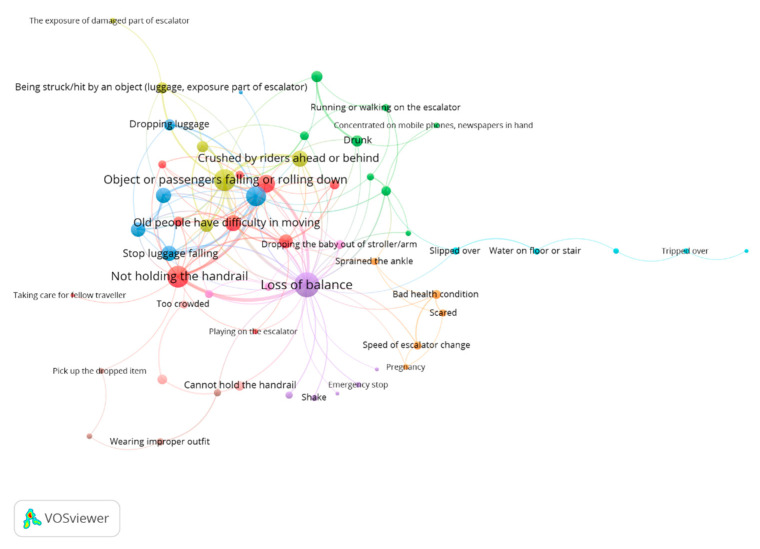
The entire hazard map (61 hazards) of the proposed CEHN.

The hazard chains in Table 2 can be integrated into a network, which can better capture the full complexity of the multiple relationships among different hazards and scenarios. The VOS Viewer software was used to assess the hazards in our CEHN. In order to map this network, the number of hazards in an escalator accident should not be less than 2. Therefore, 472 records were excluded. The remaining 327 records were using to form the CEHN for analysis. The labels and a partial map of hazards are shown in Figure 3 for clearer observation; the whole map of 61 hazards in the CEHN is shown in Figure A1 in the Appendix. The nodes are the hazards identified in Table 1. The edges are the interactions between the hazards. An input direction of a hazard means the hazard is a result, and an output direction means the hazard is a cause.

In our case, there were 61 nodes and 1015 edges in the whole network. By combining multiple edges, there were 224 edges in the network in total. The entire CEHN offers some intuitive insight into critical hazards. Hazards with more outgoing edges are more centrally located, such as “Loss of balance”, Not holding the handrail”, “Object or riders falling or rolling down”, and “Crushed by riders ahead or behind”. Hazards with fewer edges are located around the outer boundary of the CEHN, such as “Escalator floor plate bulge”, “Handrail poor electrical isolation”, and “Got electrocution from escalator”, which were detached from the entire network.

### 4.3. Analyzing the Topological Characteristics of Beijing CEHN

#### 4.3.1. Degree and Strength Distribution

**Figure 4:**
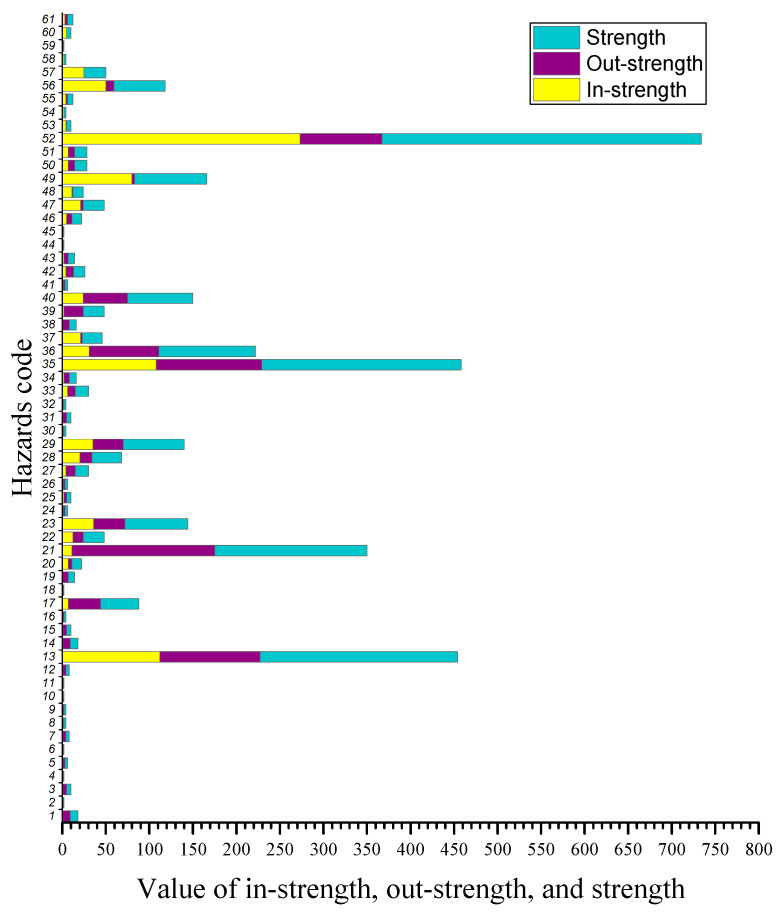
Stacked bar of in-strength, out-degree, and degree of hazards.

The in-strength, out-strength, and strength of all nodes in our CEHN are shown in Figure 4. On the whole, the average strength is 31, which means each hazard occurred 31 times on average, between 2016 to 2018. The average degree is 5, which means that each hazard has direct relationships with five other hazards in the CEHN. The results of degree and strength of hazards are different. The hazard “Loss of Balance” was the highest strength (without direction), followed by the hazard “Not holding the handrail”. These two kinds of hazards not only lead to accidents independently but also lead to accidents together with most other hazards normally, which led them to have the highest strength in the CEHN. The hazard “Object or riders falling or rolling down” was ranked third, and the hazard “Carrying bulk items (luggage)” was ranked fourth in hazard strength.

**Figure 5:**
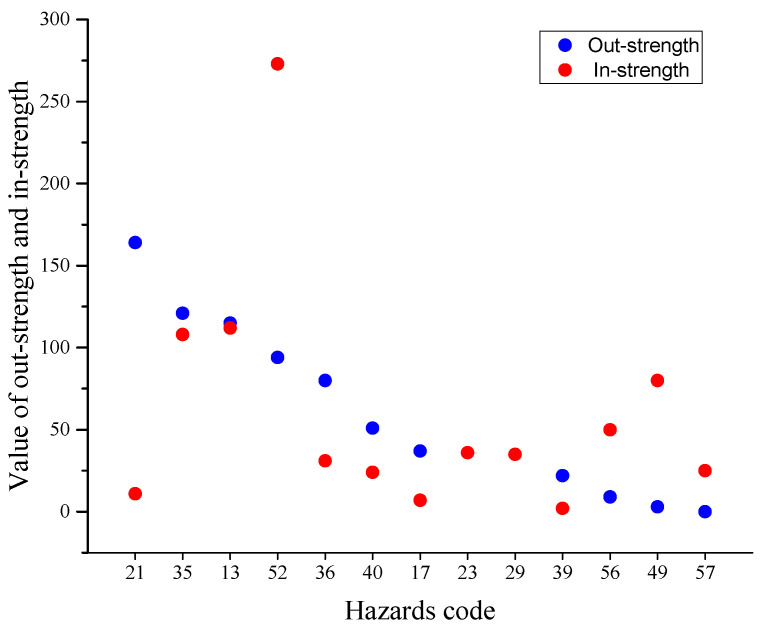
Difference between top ten ranked hazards by out-strength (blue) and in-strength (red).

The top ten ranked hazards by in-strength and out-strength are compared in Figure 5. Apparently, there were differences between them. According to the sorting, Figure 5 shows a total of 13 hazards. Because there is no sufficient space to label each vertex with text, hazards code was used as horizontal axis. The horizontal axis was ordered by out-strength of 13 hazards. When the blue ball is higher than the red ball, the hazard is a trigger hazard. On the contrary, if the red ball is higher than the blue ball, the hazard is a consequence hazard. When the blue ball is near the red ball, the hazard is a transitional hazard. For example, “Carrying bulk items (luggage)” ranked first in out-strength (with a strength of 164) but did not appear in the top 10 ranking, in terms of in-strength. This means that “Carrying bulk items (luggage)” was a trigger hazard. The hazards “Not holding the handrail”, “Object or riders falling or rolling down”, “Old people have difficulty in moving”, “Cannot lift the luggage”, and “Stop luggage falling” had almost the same rank in in-strength. These kinds of hazards were transitional hazards. “Loss of balance”, “Crushed by riders ahead or behind”, “Being pulled out of balance by suitcase”, and “Being pulled out of balance by falling riders” had higher in-strength than out-strength, which means that these hazards were consequence hazards.

#### 4.3.2. Un-Weighted and Weighted CC of Hazards

The CC could only be calculated for 48 hazards, as the other 13 hazards only had one neighbor (thus making it impossible to form a triple). Table 3 shows the top ten hazards with un-weighted and weighted CC in our CEHN. The un-weighted CC values of the hazards in the CEHN ranged from 0 to 0.8 and weighed CC values ranged from 0 to 0.002. The closer the value of CC is to 1, the stronger the accessibility of its neighbor hazards. This means that the prevention of hazards that have higher CC values does not help as much, in terms of accident prevention, as its neighboring hazards still have strong relationships.

The top ten hazards ranked by un-weighted CC and weighted CC were slightly different. From the topological structure perspective, the hazards “Dropping luggage”, “Accompanying riders”, and “Stop luggage falling” had un-weighted CC values higher than 0.5. Furthermore, the un-weighted CC values of the rest of the hazards roughly fluctuated between 0.1 to 0.5, except 12 hazards with the lowest value (0). The average un-weighted CC value (i.e., for the entire CEHN) was 0.2338. From a weighted topological structure perspective, although the order of top ten hazards was different with top ten hazards in un-weighted CEHN, most of the hazards were the same. The maximum weight on events (edges) was 107, which was the reason weighed CC values ranged from 0 to 0.002. From the result, it can be seen that the frequency of events would not change the cluster ability of hazards too much.

#### 4.3.3. Betweenness Centrality

The betweenness rankings (for hazards and hazard interactions) are presented in Table 4. According to the betweenness of hazards, the top ten hazards are all related to riders in task-driven behavior, improper riding behavior, and bad health conditions, except for the hazard “Object or riders falling or rolling down”, which belongs in the environmental cause category. “Loss of balance” had the maximum number of shortest paths passing through it. It was followed by “Object or riders falling or rolling down” and “Carrying bulk items (luggage)”, which had betweenness values 0.1607 and 0.1248, respectively. Removing these three hazards would result in partitioning of the CEHN. In other words, if “Loss of balance”, “Object or riders falling or rolling down”, or “Carrying bulk items (luggage)” are prevented, communication between the remaining hazards in the CEHN could be avoided.

Apparently, the top 3 hazard interrelations are related to “Object or riders falling or rolling down”, which are “Loss of balance” to “Object or riders falling or rolling down”, “Object or riders falling or rolling down” to “With children/attending to children”, and “Object or riders falling or rolling down” to “Carrying bulk items (luggage)”. It is clear that the prevention of “Object or riders falling or rolling down” would have more efficient effect on accident prevention, compared with prevention of “Loss of balance”. Furthermore, four edges among the top ten edges are related to “Carrying bulk items (luggage)”, which confirms the results of node betweenness in the CEHN. In addition, the other two hazard interrelations were related to “Drunk”, which had a relatively low rank in arc betweenness. This should also be considered as a key bridge, according to the results of the arc betweenness analysis.

## 5. Discussion

From the results of our analysis, it can be seen that most escalator accidents in MRT stations were the result of cascading hazards. “Crushed under ahead/back passengers”, “Being pulled out of balance by suitcase”, and “Being pulled out balance by the falling passengers” were common forms of consequence hazards. Prevention of the triggering RPB and key bridges in the cascading network could block the occurrence of transitional hazards and consequence hazards, as well as the formation of the entire CEHN.

“Carrying bulk items”, a passenger task-driven behavior, proved to be a trigger RPB and key bridge in our CEHN, with a lack of interactions among its neighboring hazards. Therefore, prevention of passengers who carry bulk items riding escalators may be efficient in reducing the occurrence of related key bridge hazards, such as “Objects falling down”, “Stepping off the escalator”, and “Quarrels with others”. “Taking care of kids” is another passenger task-driven behavior that was shown to be a trigger RPB. Although “Taking care of kids” was not a key bridge, its post hazard “With children/attention to children” was. Both RPB hazards had lower redundancy interactions among their neighbor hazards. Therefore, prevention of passengers who are “Taking care of kids” to ride escalators may be useful in blocking the occurrence of the key bridge hazard “With children/attention to children”. “Old people have difficulty in moving”, which is considered as a health condition of riders, was proved to be a trigger RPB, as well as a key bridge. Its neighbor hazards had lower hazard interactions. Therefore, it may be efficient to block the occurrence of transitional hazards and consequence hazards, such as “Loss of balance”.

Therefore, passengers who are carrying bulk items, taking care of kids, and old people who have difficulty in moving should be encouraged to take elevators. These findings are consistent with practice. Additionally, the findings also strengthen the knowledge that improper riding behavior, such as not holding the handrail, stepping off the escalator, and picking up dropped items, should be avoided as well. Our results also revealed the opposite of what is already known: Prevention of some behavior, such as dropping luggage, accompanying riders, stopping luggage falling, looking back, playing on the escalator, and using personal items on the escalator, will not effectively prevent the occurrence of cascading escalator accidents, as there are hazard interactions between the neighboring hazards of these behaviors. For example, the trigger hazard for “dropping luggage” is “carrying bulk items” and its consequence hazard is “object or passenger falling down”. Both “Carrying bulk items” and “Object or passenger falling down” are neighboring hazards of “Dropping luggage”; Objects or passengers falling down could be caused directly by carrying bulk items without dropping luggage.

However, the supply of elevators in subway stations is not sufficient, especially when the passenger flow is large. Waiting for the elevator requires a high time cost, which results in those passengers who are encouraged to take elevators being unwilling to take them. Therefore, it is worth discussing how to prevent cascading accidents if these passengers use escalators. In some sense, when one risk behavior is normal, there must be something wrong with the management design. The behaviors of riders are often determined by the management design, being the external manifestation of the internal function of the complex system. Injury prevention by controlling quite odd behavior is usually compiled into new laws or rules [16].

**Figure 6:**
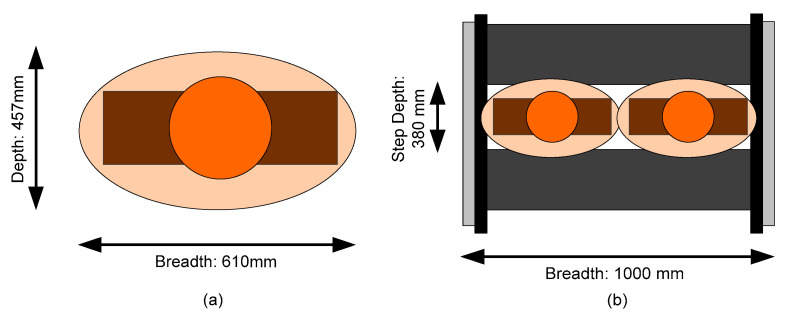
(**a**) Human ellipse; and (**b**) The effect of the human ellipse when standing side-by-side on a one-meter width escalator.

There is a well-established escalator etiquette, walking left and standing right (WLSR), which has widely been advocated for and adopted in escalator management across the world. This familiar rule allows passengers who would prefer to let the machinery do all the work relax to one side (on the right), while those in a hurry can pass by (on the left). However, according to the concept of passenger space proposed in reference [36], WLSR itself may be a source of some passenger risk behavior. Figure 6a shows the diagram of humans as ellipses, as represented by a round part (head) and a rectangular part (shoulders) for passengers; the tolerated space for a passenger (termed the human ellipse) is also indicated. The width of the escalator in many MRT stations (e.g., in Beijing and London) is 1 meter. Further, Figure 6 also shows two people standing on one step on the escalator [37]. Figure 6b shows how, when two people stand side by side, their tolerated spaces slightly overlap. It also makes it less comfortable for people to pass on escalators.

**Figure 7:**
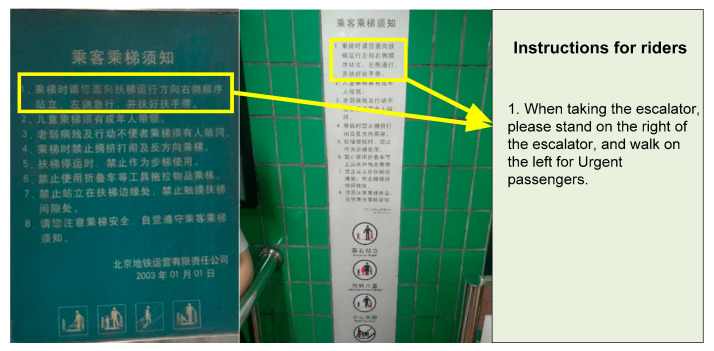
Instructions for escalator passengers at the Wudaokou and Xizhimen metro rail transit (MRT) stations in Beijing.

Unfortunately, when a passenger carries a load or does not stand near to the comb plates at the side, they may be knocked over by a passing person. Accidents related to the hazard “Carrying bulk items (luggage)” (ranking first in out-degree, 164; ranking third in node betweenness) in Beijing MRT accounted for 49.24% of cases (161 of 327 subway escalator accidents). Similar results were found for Hong Kong MTR [37]. However, in many MRTs worldwide, passengers are still encouraged to follow the WLSR rule (see Figure 7). Face-to-face interviews with senior security managers and station safety officers in Beijing subway station have been conducted about the riding rules on WLSR. Their understanding of the rule was not consistent. The senior security manager believed it was not safe to WLSR and that it should not be encouraged. However, most of the station safety officers thought it should be encouraged and that it can improve the efficiency of escalator capacities.

The total capacity for WLSR is approximately 120 people per minute (ppm) [36]. The capacity of an escalator is 108 ppm under the condition 43.2 meters per minute, two steps for one person, both sides occupied. However, the rider’s walking time is limited to them first entering the escalator. The case depicted above is for full-load condition escalators; there are also some cases with inadequate riders. The capacity of an escalator is 3700 ped/h under the rule “walk-stand mix”, but the capacity is 4400 ped/h under the rule “no climbing allowed” [19].

**Figure 8:**
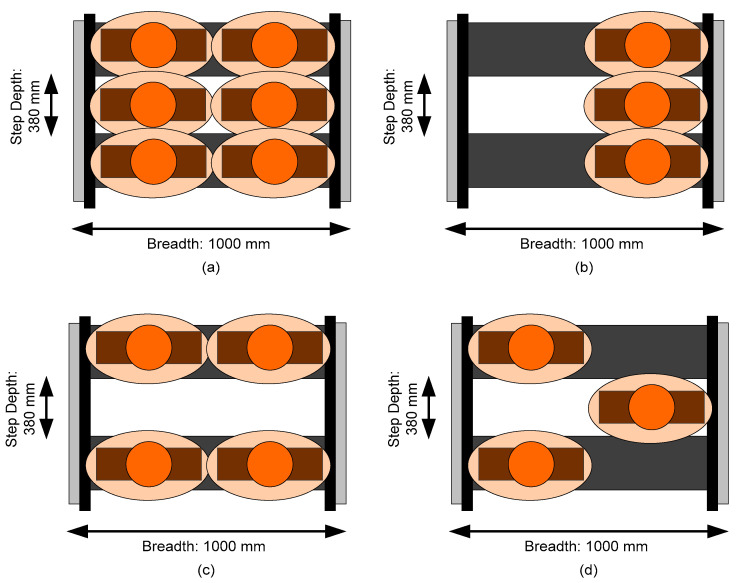
The effect of the human ellipse in four types of standing: (**a**) standing side-by-side and standing directly in front of one another; (**b**) standing directly in front of each other at the right side; (**c**) standing side-by-side and leaving one empty step in front; and (**d**) one person standing per step and alternately left and right.

Therefore, walking on escalators does not always improve the flow of escalators, especially in crowded conditions. Riders should not be encouraged to walk on escalators during rush hours and, in an emergency scenario, walking on escalators should be prohibited as it reduces variability in the system and increases flow [19,38]. Therefore, how should passengers stand on escalators? Firstly, in order to maximize the capacity of escalator transportation, passengers are encouraged to stand side-by-side and directly in front of one another (as shown in Figure 8a). However, people tend to avoid space occupied by others if they stand directly in front of them. Once a passenger is “Carrying bulk items (luggage)”, the shoulder overlap of two passengers in the same step becomes bigger. In this case, the RPB “Loss of balance” is likely to happen, due to insufficient standing space. Certainly, this type of standing should be avoided. Two people standing directly in front of each other is understandable, but three people doing the same is strange, unless they know each other; see Figure 8b. Additionally, standing close to each other (e.g., directly in front of each other on an escalator), may be the reason for the RPB “Crushed by riders ahead or behind”, which comprised 16.5% of accidents (83 cases; ranked sixth in degree distribution) in our collected accident data.

Therefore, the riders who are standing on the escalator should leave one step for each other. This means that there is one step space, on average, for the people in the escalator; see Figure 8c. The transportation occupancy in this situation is the same as with passengers standing one person per step and alternately left and right, as shown in Figure 8d. When considering both safety and the comfort of passengers, it is better to encourage passengers to stand one per step and alternately left and right. The first reason for this is that the human ellipse would be larger when a passenger is carrying a load which will increase their overlap of personal space. The larger the overlap, the more often collision accidents happen. The second reason is that, in our results, it was proved that “Being pulled out of balance by falling riders” (ranked ninth in in-degree, ranked 23rd in node betweenness) was a consequence of the falling of side passengers. If passengers stand alternately left and right, this type of accidents can be avoided or, at least, the adverse effects of them can be reduced. The third reason is that “passengers standing one per step and alternately left and right” is friendly to people with disabilities when they cannot hold the handrail with their right hand. Additionally, it is easy to hold the handrail on both sides in an emergency.

## 6. Conclusions

The current study provides evidence that through combination of FTDM accident investigation model and complex network analysis method, critical and non-critical RPB and OH-RPB in a complicated cascading hazards network can be identified. Through the discussion of the case study, it can be seen that the results are consistent in critical and non-critical RPB and OH-RPB. Prevention of critical RPB can block the formation of cascading accident networks. The method can be used by safety managers to make the corresponding preventive measures according to the results in daily management. The findings can guide the allocation of limited preventive resources to critical hazards rather than non-critical hazards. Moreover, the defects of management plan and product design can be re-examined according to the research results.

Last of all, we want to note two drawbacks of the study. Firstly, 472 of the data we collected in the case study had an insufficient process description where only two hazards could be extracted. These data were not used in the establishing of CEHN, which may change the topological structure if these data were used. The results of the topological structure and function on the topological structure of CEHN highly depend on the accuracy of the cascading accident process description. Besides, the proposed method was only applied to Beijing MRT stations; a future study should apply the proposed method to other cities to explore whether generalized results exits between different cases. Secondly, only degree, strength, clustering coefficient, and betweenness characteristics were used for CEHN analysis. In the future study, spatial autocorrelation analysis should be considered. As most hazards were the result of domino effect of other hazards, each hazard corresponds to a specific spatial attribute, including geographical location, demographic attributes, regional morphology, economic level, etc. It is a really interesting question whether there are spatial autocorrelation characteristics of CEHN.

## Figures and Tables

**Table 1:** Hazards identified in 327 accidents.

Code Hazards Description	Code Hazards Description
***Escalator***	***Rider (Improper riding behavior–non-task-active)***
1 Speed of escalator change	30 Pick up the dropped item
2 Handrail poor electrical isolation	31 Wearing improper outfit
3 Handrails are not synchronized with the escalator	32 Drop the clothing items
4 Insufficient height between escalator and the Billboard above	33 Stepping off the escalator
5 Emergency stops	34 Step on edges of escalator steps
6 Unexpected reverse	35 Not holding the handrail
7 Shake	36 Not standing still
***Maintenance***	***Rider (Bad health condition)***
8 Water absorbing blanket bulge	37 Fainting/black out
9 The exposure of damaged part of escalator	38 Use of personal items on the escalator (e.g., pushchair, crutch)
10 The camouflage door under the escalator opens	39 Drunk
11 Escalator floor plate bulges	40 Old people have difficulty in moving
***Environment***	41 Pregnancy
12 Water on floor or steps	42 Small kids unbalanced
13 Object or riders falling or rolling down	43 Lack of lower body mobility in older/unhealthy people
14 Too crowded	44 Poor eyesight
*Rider (task-driven behavior)*	45 Disabled
15 Running or walking on the escalator	46 Bad health condition
16 Concentrated on mobile phones, newspapers in hand	*Rider (Last passive action)*
17 With children/attending to children	47 Being struck/hit by luggage
18 Taking care for fellow traveler	48 Being knocked over by riders
19 Carrying wheelchair or trolley or bicycle	49 Crushed by riders ahead or behind
20 Dropping the baby out of stroller/arm	50 Cannot hold the handrail
21 Carrying bulk items (luggage)	51 Cannot stand still
22 Dropping luggage	52 Loss of balance
23 Cannot lift the luggage	53 Slipped over
24 Playing on the escalator	54 Tripped over
25 Quarrels with others	55 Entrapment of outfit
26 Looking back	56 Being pulled out of balance by suitcase
27 Accompanying riders	57 Being pulled out of balance by falling riders
28 Help the falling riders	58 Being Knocked over/hit by the part of escalator
29 Stop luggage falling	59 Got electrocution from escalator
	60 Scared
	61 Sprained the ankle

**Table 2:** Hazard chains of 327 escalator accidents.

Case No.	Hazard Chains	Case No.	Hazard Chains	Case No.	Hazard Chains
1	27→36→52→13→47→57	110	36→52→13→49	219	21→35→52
2	27→36→52→13→47→57	111	36→52→13→49	220	21→35→52
3	27→36→52→13→48→57	112	40→52→28→49	221	21→35→52
4	27→36→52→13→49→57	113	13→21→52→49	222	21→35→52
5	42→52→13→17→49→57	114	21→35→52→49	223	21→35→52
6	21→22→56→13→49	115	21→35→52→49	224	21→35→52
7	17→39→36→52→49	116	21→35→52→49	225	21→35→52
8	21→29→23→56→52	117	21→35→52→49	226	21→35→52
9	37→21→22→13→28→57	118	21→35→52→49	227	21→35→52
10	38→35→36→13→28→57	119	17→42→52→49	228	21→35→52
11	21→23→52→13→28→57	120	17→36→20→52	229	21→35→52
12	21→29→52→13→28→57	121	38→40→21→52	230	21→35→52
13	17→35→52→13→28→57	122	21→25→26→52	231	21→35→52
14	17→35→52→13→28→57	123	40→13→28→52	232	21→35→52
15	21→35→52→13→28→57	124	36→52→28→52	233	21→35→52
16	17→35→52→13→47	125	19→35→29→52	234	21→35→52
17	17→40→52→13→49	126	15→25→33→52	235	21→35→52
18	17→42→52→13→49	127	14→21→35→52	236	21→35→52
19	21→23→56→13→49	128	14→21→35→52	237	21→35→52
20	21→23→56→13→49	129	33→21→35→52	238	21→35→52
21	21→23→56→13→49	130	38→21→35→52	240	21→35→52
22	21→23→56→13→49	131	45→21→35→52	241	21→35→52
23	21→29→56→13→49	132	21→23→35→52	242	21→35→52
24	21→29→56→13→49	133	21→23→35→52	243	21→35→52
25	21→29→56→13→49	134	21→23→35→52	244	21→35→52
26	21→22→13→17→49	135	21→23→35→52	245	21→35→52
27	39→37→13→17→49	136	21→23→35→52	246	21→35→52
28	17→40→52→20→49	137	40→27→35→52	247	21→35→52
29	17→40→52→20→49	138	13→28→35→52	248	21→35→52
30	17→40→52→20→52	139	13→28→35→52	249	21→35→52
31	17→42→21→23→52	140	21→29→35→52	250	21→35→52
32	21→29→40→23→56	141	21→40→35→52	251	21→35→52
33	21→29→40→23→56	142	43→17→36→52	252	21→35→52
34	36→52→13→28→56	143	21→35→40→52	253	21→35→52
35	36→52→13→28→57	144	21→35→40→52	254	21→35→52
36	36→52→13→28→57	145	21→35→40→52	255	21→35→52
37	21→35→52→28→57	146	21→35→40→52	256	21→35→52
38	31→32→30→35→57	147	21→35→40→52	257	21→35→52
39	21→22→13→47→57	148	17→40→42→52	258	21→35→52
40	21→22→13→47	149	14→51→50→52	259	21→35→52
41	21→22→13→47	150	14→51→50→52	260	21→35→52
42	21→22→13→47	151	14→51→50→52	261	21→35→52
43	21→22→13→47	152	14→51→50→52	262	21→35→52
44	21→22→13→47	153	14→51→50→52	263	21→35→52
45	21→22→13→47	154	14→51→50→52	264	21→35→52
46	21→22→13→47	155	14→51→50→52	265	21→35→52
47	21→29→13→47	156	14→51→50→52	266	21→35→52
48	21→29→13→47	157	21→22→13→56	267	21→35→52
49	21→29→13→47	158	21→29→13→56	268	21→35→52
50	21→29→13→47	159	21→29→13→56	269	21→35→52
51	35→52→13→48	160	21→29→13→56	270	24→35→52
52	35→52→13→48	161	21→29→13→56	271	38→35→52
53	36→52→13→48	162	21→40→23→56	272	40→35→52
54	36→52→13→48	163	21→40→29→56	273	38→36→52
55	36→52→13→48	164	21→23→35→56	274	40→36→52
56	36→52→13→48	165	21→23→35→56	275	40→36→52
57	36→52→13→48	166	21→29→40→56	276	40→36→52
58	17→40→52→48	167	21→29→40→56	277	40→36→52
59	21→29→13→49	168	40→36→13→57	278	40→36→52
60	21→29→13→49	169	40→36→13→57	279	40→36→52
61	19→34→13→49	170	40→36→13→57	280	40→36→52
62	19→34→13→49	171	36→52→13→57	281	40→36→52
63	17→36→13→49	172	36→52→13→57	282	40→36→52
64	35→36→13→49	173	36→52→28→57	283	42→36→52
65	40→36→13→49	174	40→52→28→57	284	43→36→52
66	35→52→13→49	175	40→52→28→57	285	43→36→52
67	35→52→13→49	176	40→52→28→57	286	16→39→52
68	35→52→13→49	177	40→52→28→57	287	21→40→52
69	35→52→13→49	178	40→52→28→57	288	38→43→52
70	35→52→13→49	179	40→52→28→57	289	38→43→52
71	35→52→13→49	180	40→27→36→57	290	1→46→52
72	35→52→13→49	181	1→46→61→60	291	41→53→52
73	35→52→13→49	182	17→52→20	292	33→53→52
74	35→52→13→49	183	40→35→36	293	31→55→52
75	35→52→13→49	185	21→13→47	294	31→55→52
76	36→52→13→49	186	21→13→47	295	21→61→52
77	36→52→13→49	187	21→13→47	296	33→61→52
78	36→52→13→49	188	36→52→49	297	12→8→54
79	36→52→13→49	189	42→17→52	298	19→13→56
80	36→52→13→49	190	42→17→52	299	21→23→56
81	36→52→13→49	191	42→17→52	300	21→23→56
82	36→52→13→49	192	35→21→52	301	21→23→56
83	36→52→13→49	193	38→21→52	302	21→23→56
84	36→52→13→49	194	17→24→52	303	21→23→56
85	36→52→13→49	195	21→29→52	304	21→23→56
86	36→52→13→49	196	21→29→52	305	21→23→56
87	36→52→13→49	197	21→29→52	306	21→23→56
88	36→52→13→49	198	15→33→52	307	21→23→56
89	36→52→13→49	199	15→33→52	308	21→23→56
90	36→52→13→49	200	16→33→52	309	21→23→56
91	36→52→13→49	201	21→33→52	310	21→23→56
92	36→52→13→49	202	21→35→52	311	21→23→56
93	36→52→13→49	203	17→35→52	312	21→23→56
94	36→52→13→49	204	17→35→52	313	21→23→56
95	36→52→13→49	205	17→35→52	314	21→29→56
96	36→52→13→49	206	17→35→52	315	21→29→56
97	36→52→13→49	207	17→35→52	316	21→29→56
98	36→52→13→49	208	17→35→52	317	21→29→56
99	36→52→13→49	209	17→35→52	318	21→29→56
100	36→52→13→49	210	17→35→52	319	21→29→56
101	36→52→13→49	211	17→35→52	320	21→29→56
102	36→52→13→49	212	17→35→52	321	21→29→56
103	36→52→13→49	213	18→35→52	322	21→29→56
104	36→52→13→49	214	19→35→52	323	27→35→57
105	36→52→13→49	215	21→35→52	324	27→35→57
106	36→52→13→49	216	21→35→52	325	1→41→60
107	36→52→13→49	217	21→35→52	326	1→46→60
108	36→52→13→49	218	21→35→52	327	1→46→60
109	36→52→13→49		21→35→52		1→46→60

**Table 3:** Top ten hazards by Clustering coefficient.

Rank	Hazard	Un-Weighted Clustering Coefficient	Hazard	Weighted Clustering Coefficient
1	Dropping luggage	0.83	Dropping luggage	2.01 × 10^−3^
2	Accompanying riders	0.67	Not standing still	9.84 × 10^−4^
3	Stop luggage falling	0.57	Cannot lift the luggage	9.45 × 10^−4^
4	Looking back	0.50	Accompanying riders	7.68 × 10^−4^
5	Playing on the escalator	0.50	Stop luggage falling	7.32 × 10^−4^
6	Cannot lift the luggage	0.50	Object or riders falling or rolling down	7.01 × 10^−4^
7	Use of personal items on the escalator (e.g., pushchair, crutch)	0.50	Help the falling riders	6.64 × 10^−4^
8	Stepping off the escalator	0.50	Looking back	5.76 × 10^−4^
9	Small kids unbalanced	0.45	Playing on the escalator	4.39 × 10^−4^
10	Being pulled out of balance by suitcase	0.43	Use of personal items on the escalator (e.g., pushchair, crutch)	3.23 × 10^−4^

**Table 4:** Top ten hazards and hazard interactions ranked by the betweenness.

Rank	Hazards	Value	Hazard Interactions	Value
1	Loss of balance	0.1663	Loss of balance-Object or riders falling or rolling down	0.3798
2	Object or riders falling or rolling down	0.1607	Object or riders falling or rolling down–With children/attending to children	0.2147
3	Carrying bulk items (luggage)	0.1248	Object or riders falling or rolling down Carrying bulk items (luggage)	0.1403
4	With children/attending to children	0.0641	With children/attending to children-Drunk	0.0744
5	Not holding the handrail	0.0506	Not holding the handrail-Carrying bulk items (luggage)	0.0713
6	Stepping off the escalator	0.0241	Carrying bulk items (luggage)–Quarrels with others	0.0674
7	Old people have difficulty in moving	0.0219	Carrying bulk items (luggage)–Stepping off the escalator	0.0636
8	Drunk	0.0134	Carrying bulk items (luggage)–Sprained the ankle	0.0605
9	Quarrels with others	0.0128	Pick up the dropped item–Not holding the handrail	0.0558
10	Pick up the dropped item	0.0121	Drunk-Fainting/black out	0.0512
